# Patient-Reported Characteristics Across Dual-Eligible Medicare Advantage Plan Types

**DOI:** 10.1001/jamanetworkopen.2025.5791

**Published:** 2025-04-18

**Authors:** Kendra Offiaeli, David J. Meyers, Eliza Macneal, Kenton J. Johnston, Brittany Brown-Podgorski, Eric T. Roberts

**Affiliations:** 1 Brown University School of Public Health, Providence, Rhode Island; 2 Center for Advancing Health Policy Through Research, Brown University, Providence, Rhode Island; 3 University of Pennsylvania Perelman School of Medicine, Philadelphia; 4 Leonard Davis Institute of Health Economics, University of Pennsylvania, Philadelphia; 5 Washington University in St Louis School of Medicine, St Louis, Missouri; 6 University of Pittsburgh School of Public Health, Pittsburgh, Pennsylvania

## Abstract

**Importance:**

A growing proportion of full-benefit dual-eligible Medicare and Medicaid beneficiaries (ie, individuals receiving Medicare and full Medicaid) are enrolled in Medicare Advantage (MA). MA plans vary in capacity and incentives to coordinate Medicare and Medicaid services, but little is known about the characteristics of dual-eligible beneficiaries across plan types.

**Objectives:**

To compare the health and demographic characteristics of full-benefit dual-eligible beneficiaries across 4 MA plan types varying in Medicare-Medicaid service coordination and spending requirements.

**Design, Setting, and Participants:**

This cross-sectional study analyzed Medicare Health Outcomes Survey (HOS) data from 2017 to 2019, including 147 923 full-benefit dual-eligible beneficiaries (mean response rate, 29%) enrolled in MA in January of the survey year. Analyses were conducted from January 2024 to January 2025.

**Exposure:**

Enrollment in different MA plans.

**Main Measures and Outcomes:**

This study examined beneficiary characteristics and enrollment across 4 MA plan types: standard MA plans serving dual and nondual beneficiaries; coordination-only dual-eligible Special Needs Plans (D-SNPs) with Medicaid contracts and care coordination requirements serving dual-eligibles; D-SNP look-alikes, marketed to dual-eligible beneficiaries but exempt from D-SNP regulations; and fully integrated D-SNPs (FIDE-SNPs), which integrate Medicare and Medicaid services and spending. Beneficiary characteristics included demographics, self-reported comorbidities, functional status, living arrangements, and community-level factors, like the Area Deprivation Index (ADI).

**Results:**

The study included 147 923 dual-eligible beneficiaries with full Medicaid (mean (SD) age, 67.7 [13.9] years; 93 803 [63.4%] female), distributed across FIDE-SNPs (25 755 beneficiaries), coordination-only D-SNPs (65 220 beneficiaries), D-SNP look-alike plans (5193 beneficiaries), and standard MA plans (51 755 beneficiaries). Overall, 14 215 respondents (9.6%) were aged 85 years or older, and 9618 respondents (6.5%) lived with a caregiver. Respondents had a mean (SD) of 4.2 (2.7) comorbidities and 2.3 (2.8) difficulties with activities of daily living (measured on a 0-12 scale). FIDE-SNPs enrolled more beneficiaries aged 85 years or older (eg, FIDE-SNP: 59.0% [95% CI, 58.0%-59.9%]; coordination-only D-SNP: 16.1% [95% CI, 15.3%-16.8%]), living with a caregiver (eg, FIDE-SNP: 56.6% [95% CI, 55.3%-58.0%]; coordination-only SNP: 15.3% [95% CI, 14.3%-16.2%]), and FIDE-SNP beneficiaries had more comorbidities (mean [SD], FIDE-SNP: 4.6 [2.7]; coordination-only SNP: 4.1 [2.7]) and higher scores of difficulties with activities of daily living (mean [SD], FIDE-SNP: 3.1 [3.1]; coordination-only SNP: 2.1 [2.6]) vs beneficiaries with other plan types. In counties offering FIDE-SNPs, 38.4% (95% CI, 37.5%-39.3%) of individuals in areas with the greatest socioeconomic disadvantage were enrolled in coordination-only D-SNPs and 41.3% (95% CI, 40.2-42.4) were enrolled in FIDE-SNPs. In areas with the least socioeconomic disadvantage, 32.0% (95% CI, 31.3%-32.8%) of respondents were enrolled in coordination-only D-SNPs and 43.2% (95% CI, 42.4%-43.9%) respondents were enrolled in FIDE-SNPs. Most differences remained in multivariate analyses.

**Conclusions and Relevance:**

In this cross-sectional study of full-benefit dual-eligible beneficiaries, substantial health burdens were reported, with notable differences across plan types. These findings highlight the need for tailored interventions to improve care and further research to understand MA plan selection in this population.

## Key Points

**Question:**

How do the characteristics of dual-eligible beneficiaries with full Medicaid vary across Medicare Advantage (MA) plans that attain different levels of coordination and integration with Medicaid?

**Findings:**

This cross-sectional study including 147 923 dual-eligible beneficiaries found differences in the characteristics of full-benefit dual-eligible beneficiaries across MA plan types. Older dual-eligible individuals and those with multiple functional limitations were more likely and those in highly socioeconomically disadvantaged neighborhoods were less likely to enroll in fully integrated dual-eligible Special Needs Plans compared with less integrated plan types.

**Meaning:**

There are notable health and demographic differences among dual-eligible beneficiaries across MA plans with varying levels of Medicaid coordination and integration.

## Teaser

This cross-sectional study compares the health and demographic characteristics of full-benefit dual-eligible beneficiaries across 4 Medicare Advantage plan types varying in Medicare-Medicaid service coordination and spending requirements.

## Introduction

Enrollment in Medicare Advantage (MA) among full-benefit dual-eligible beneficiaries—individuals eligible for and enrolled in both Medicare and full Medicaid—has increased, mirroring the broader Medicare population.^1,2^ MA, the managed care alternative to traditional Medicare, is administered by private insurers that receive risk-adjusted, capitated payments to provide Medicare-covered services to enrollees. In 2019, 36% of full-benefit dual-eligible beneficiaries were enrolled in MA,^3^ and as of 2022, nearly 50% of dual-eligible beneficiaries with full Medicaid were enrolled in MA.^4^

Full-benefit dual-eligible beneficiaries may enroll in different MA plan types, varying in their responsibility to coordinate care with Medicaid and managing spending.^5,6,7^ First, standard MA plans manage only Medicare-covered services (outpatient care, acute and postacute care, and prescription drugs), do not coordinate care with Medicaid, and may enroll dual-eligible and non–dual-eligible beneficiaries. Second, dual-eligible Special Needs Plans (D-SNPs), exclusively enroll dual-eligible beneficiaries, and must have contracts and coordinate care with state Medicaid programs. D-SNPs may also manage some Medicaid-funded services directly.^8^ Third, D-SNP look-alike plans are standard MA plans marketed toward and primarily composed of dual-eligible beneficiaries, yet lack the D-SNP regulations of Medicaid contracts and care coordination.^9^ Fourth, fully integrated D-SNPs (FIDE-SNPs) are a subset of D-SNPs that have capitation contracts for Medicaid-covered long-term care, and usually, behavioral health spending.

FIDE-SNPs are financially integrated care plans because either the plan or the parent company operating a companion Medicaid managed care plan bears risk for both Medicare and Medicaid spending. Financial integration establishes an incentive for plans to coordinate the full range of Medicare- and Medicaid-funded care for beneficiaries.^6,10,11^ Prior to 2021—the period this study covers—D-SNPs were classified by the Centers for Medicare & Medicaid Services (CMS) as either FIDE-SNP or non–FIDE-SNP plans. Beginning in 2021, CMS began to categorize D-SNPs into 1 of 3 groups: FIDE-SNPs (largely financially integrated with Medicaid), HIDE-SNPs (partially financially integrated with Medicaid), or coordination-only D-SNPs (minimally financially integrated). HIDE-SNPs were not available during the study period (2017-2019). Other integrated care plans include the Program of All-inclusive Care for the Elderly and Medicare-Medicaid Plans, which were phased out by 2025, and are not included in this analysis.

Distinctions between MA plans with different levels of Medicaid coordination and financial integration have drawn increased attention by policymakers pursuing insurance reforms for the dual-eligible population. They focus on increasing coordination across Medicare and Medicaid and expanding enrollment in integrated care plans (eg, FIDE-SNPs).^12,13,14^ In addition, policymakers seek to address detractors to integrated care, including the growth of D-SNP look-alike plans.^9^ However, little is known about the characteristics of dual-eligible beneficiaries who enroll in different MA plan types, including health, demographic, and social factors that influence the need for care coordination.^15,16^ In this study, we used national survey data to examine the characteristics of dual-eligible beneficiaries enrolled in different MA plan types. We had 2 objectives: first, to describe the characteristics of dual-eligible beneficiaries enrolled in different MA plan types nationally, and second, in counties where at least 1 FIDE-SNP—the most integrated plan type—was offered, to examine which beneficiary characteristics were associated with enrollment in FIDE-SNPs vs other plan types.

## Methods

This cross-sectional study was deemed exempt from review and informed consent by the Brown University institutional review board because data were deidentified. This study is reported following the Strengthening the Reporting of Observational Studies in Epidemiology (STROBE) reporting guideline.

### Data and Sample

We analyzed national data from the 2017 to 2019 Medicare Health Outcomes Survey (HOS). The HOS captures respondent-reported health, demographics, and social characteristics, exceeding the level of detail of most other datasets for this population.^17^ Each year, HOS uses random sampling to survey a representative group of beneficiaries enrolled in Medicare Advantage Organizations, with at least 500 enrollees for a baseline survey. A follow-up survey is administered 2 years later. This study examined baseline survey responses among full-benefit dual-eligible beneficiaries enrolled in MA in January of the survey year (84% of full-benefit dual-eligible beneficiaries maintained plan enrollment in the survey year). The HOS does not include survey weights to adjust for nonresponse. The response rate among full-benefit dual-eligible beneficiaries was 29%. We used the Medicare Beneficiary Summary File (MBSF), linked to the HOS at the beneficiary-year level, to identify dual-eligible MA enrollees and enrollment in specific plan types.

CMS defines D-SNPs as MA SNPs available to Medicare beneficiaries dually eligible for either full-benefit Medicaid (which covers all Medicaid services, including long-term care) or partial-benefit Medicaid (which pays for Medicare premiums and, in some cases, cost-sharing).^18^ We focused on full-benefit dual-eligible beneficiaries, who comprise 73% of the dual-eligible population, because policymakers have prioritized this group for enrollment in integrated care plans.^19^

### Identification of MA Plan Types

We used the MBSF to categorize dual-eligible beneficiaries based on enrollment in 1 of 4 mutually exclusive plan types: (1) coordination-only D-SNPs, (2) FIDE-SNPs, (3) standard MA plans, or (4) D-SNP look-alike plans. Beneficiaries were classified based on enrollment data for January of the survey year. FIDE-SNPs and coordination-only D-SNPs were identified from annual SNP reports published by CMS.^18^ For this analysis, coordination-only D-SNPs were defined as D-SNPs without a FIDE designation. We used 100% Medicare enrollment data from the MBSF to classify standard MA plans with 50% or more dual-eligible enrollees in January of the survey year as D-SNP look-alikes. Although the current threshold defined by CMS regulations for D-SNP look-alike plans stands at 70%, it is scheduled to decrease to 60% in 2026. Additionally, analysts have advocated for lowering the threshold to 50% to better capture D-SNP look-alike plans.^20^ The remaining MA plans were classified as standard MA plans. We excluded enrollees of Program of All-inclusive Care for the Elderly plans, who are included in a separate HOS survey, and those in Medicare-Medicaid Plans, institutional SNPs (I-SNPs), and chronic condition SNPs (C-SNPs), which were outside the scope of this study.

### Covariates

From the HOS, we included self-reported sociodemographic and health measures, including race, ethnicity, education, cohabitation status (ie, living alone or with a caregiver), language spoken at home, general health status, history of chronic disease, and summary scores characterizing functional status, physical health, and mental health. We categorized respondents based on self-identified nonmutually exclusive race (Asian, Black or African American, Native American, Pacific Islander, and/or White) and ethnicity (Hispanic or not Hispanic), including separate variables to capture both dimensions of identity for individuals who selected multiple categories. Race and ethnicity were included in the analysis as significant determinants of health care access and outcomes, providing essential context for understanding differences in plan characteristics. We characterized cohabitation status with 2 binary indicators identifying whether individuals reported living alone or with a paid caregiver. In the HOS, living alone and living with a paid caregiver are treated as independent binary questions, asked alongside 3 other nonmutually exclusive living arrangement questions, allowing respondents to select multiple options contributing to variation in response totals across populations. We measured disease burden through separate indicators for whether a respondent reported ever being diagnosed with hypertension or high blood pressure, congestive heart failure, stroke, diabetes, depression, pulmonary disease (emphysema, asthma, or chronic obstructive pulmonary disease), and cancer. Respondents reported general health as excellent, very good, good, fair, or poor.

The HOS incorporates the Veterans RAND 12-item Health Survey to measure health-related quality of life and disease burden. The Veterans RAND 12-item Health Survey includes 12 survey items summarized into 2 scores: the Physical Component Summary and the Mental Component Summary, both of which were used to determine quintiles based on the annual HOS population.^21,22^ The HOS also includes 6 questions about activities of daily living (ADLs), which we used to construct a summary score ranging from 0 to 12, for which higher values reflect decreased functional status. We constructed quintiles of the Physical Component Summary, Mental Component Summary, and ADL scores, based on the distribution of those scores in our sample, to categorize respondents by health and functional status. We used the MBSF for data on age, sex, and original Medicare entitlement (age, disability, or end-stage kidney disease) and linked beneficiaries to 2 area-level variables: the 2020 Area Deprivation Index (ADI), ranking 9-digit zip codes by socioeconomic disadvantage (categorized into national quintiles and deciles based on the national distribution) using the American Community Survey,^23^ and county-level residence in a physician shortage area from the Area Health Resources Files.

### Statistical Analysis

We conducted 3 analyses using beneficiary-year–level data from 2017 to 2019. First, we compared the characteristics of full-benefit dual-eligible HOS respondents across MA plan types. Second, because beneficiary enrollment decisions depend on the set of available plans and FIDE-SNPs were offered only in certain counties, we re-examined beneficiary characteristics in a geographic subsample of respondents in counties where at least 1 FIDE-SNP was offered in the survey year. FIDE-SNPs were available in 328 counties in 2019, compared with D-SNPs, offered in 1544 counties, and standard MA plans, offered in 3129 counties. Furthermore, 227 counties offering FIDE-SNPs (69.2%) also offered at least 1 D-SNP.

Third, in this geographic subsample, we used multinomial logistic regression to estimate the probability of enrollment in each plan type. We first ran bivariate models that examined each characteristic individually (ie, 1 characteristic per model) and then estimated a multivariate model that included all characteristics. Due to collinearity, ADI was excluded from the multivariate models, but bivariate associations by plan type were reported. Bivariate analyses describe how beneficiary characteristics are associated with the probability of enrollment in different plan types, and the multivariate analysis describes these associations, adjusting for all covariates. Both bivariate and multivariate models controlled for state-fixed effects to account for state differences in Medicaid eligibility. We did not adjust for county-fixed effects because such models did not converge. Analyses were conducted from January 2024 to January 2025 using Stata MP software version 18.0 (StataCorp). *P* values were 2-sided, and statistical significance was set at *P* < .05.

## Results

### Sample Characteristics and Differences Across Plan Types

The overall sample included 147 923 full-benefit dual-eligible respondents (mean (SD) age, 67.7 [13.9] years; 93 803 [63.4%] female) (Table 1). Among these, 14 215 respondents (9.6%) were aged 85 years or older. By race and ethnicity, 11 593 respondents (7.8%) self-identified as Asian, 34 927 as Black or African American (23.6%), 36 037 as Hispanic (24.4%), 7262 as Native American (4.9%), and 82 851 as White (56.0%); 16 883 respondents (11.4%) were missing data on race. The mean (SD) ADL score was 2.3 (2.8), and substantial proportions of respondents had chronic conditions, including hypertension (100 262 respondents [67.8%]), diabetes (54 118 respondents [36.6%]), depression (61 273 respondents [41.4%]), and emphysema, asthma, or chronic obstructive pulmonary disease (41 588 respondents [28.1%]).

**Table 1.** Respondent Characteristics for Overall Sample of Dual-Eligible Beneficiaries by MA Plan Type, 2017-2019

Characteristic	Respondents, No. (%)				
	All plan types (n = 147 923)	Standard MA (n = 51 755)	D-SNP look-alike (n = 5193)	Coordination-only D-SNP (n = 65 220)	FIDE-SNP (n = 25 755)
Age, y					
Mean (SD)	67.7 (13.9)	67.7 (14.2)	69.1 (12.0)	65.2 (13.7)	73.9 (12.1)
<65	47 873 (32.4)	17 303 (33.4)	1275 (24.6)	25 538 (39.2)	3757 (14.6)
65-74	54 604 (36.9)	18 417 (35.6)	2345 (45.2)	24 529 (37.6)	9313 (36.2)
75-84	31 231 (21.1)	10 635 (20.6)	1181 (22.7)	11 282 (17.3)	8133 (31.6)
≥85	14 215 (9.6)	5400 (10.4)	392 (7.6)	3871 (5.9)	4552 (17.7)
Sex					
Female	93 803 (63.4)	31 922 (61.7)	3122 (60.1)	41 197 (63.2)	17 562 (68.2)
Male	54 120 (36.6)	19 833 (38.3)	2071 (39.9)	24 023 (36.8)	8193 (31.8)
Hispanic ethnicity^a^	36 037 (24.4)	11 237 (21.7)	2392 (46.1)	15 413 (23.6)	6995 (27.2)
Race					
Asian^b^	11 593 (7.8)	3872 (7.5)	669 (12.9)	5413 (8.3)	1639 (6.4)
Black or African American	34 927 (23.6)	14 196 (27.4)	797 (15.4)	15 682 (24.0)	4252 (16.5)
Native American	7262 (4.9)	2385 (4.6)	230 (4.4)	3643 (5.6)	1004 (3.4)
Pacific Islander^c^	2511 (1.7)	544 (1.1)	55 (1.1)	1435 (2.2)	477 (1.9)
White	82 851 (56.0)	28 053 (54.2)	2740 (52.8)	36 125 (55.4)	15 933 (61.9)
Missing	16 883 (11.4)	5428 (10.5)	934 (18.0)	7044 (10.8)	3477 (13.5)
Education level					
≤Eighth grade	30 413 (20.6)	9913 (19.2)	1420 (27.3)	12 780 (19.6)	6300 (24.5)
Some high school, but did not graduate	26 091 (17.6)	9138 (17.7)	869 (16.7)	12 211 (18.7)	3873 (15.0)
High school graduate or GED	5479 (30.8)	16 081 (31.1)	1261 (24.3)	20 787 (31.9)	7350 (28.5)
Some college or 2-y degree	27 257 (18.4)	10 012 (19.3)	869 (16.7)	11 872 (18.2)	4504 (17.5)
4-y College graduate	6569 (1.4)	2538 (4.9)	292 (5.6)	2524 (3.9)	1215 (4.7)
>4-y College degree	4563 (3.1)	1725 (3.3)	163 (3.1)	1696 (2.6)	979 (3.8)
Missing	7551 (5.1)	2348 (4.5)	319 (6.1)	3350 (5.1)	1534 (6.0)
Original reason for Medicare entitlement					
Age	75 607 (51.1)	26 401 (51.0)	3205 (61.7)	29 631 (45.4)	16 370 (63.6)
Disability	71 588 (48.4)	25 000 (48.3)	1979 (38.1)	35 310 (54.1)	9299 (36.1)
ESKD	728 (0.5)	354 (0.7)	NR	279 (0.4)	86 (0.3)
Lives alone	59 964 (40.5)	19 793 (38.2)	1806 (34.8)	26 760 (41.0)	11 605 (45.1)
Lives with caregiver	9618 (6.5)	3086 (6.0)	206 (4.0)	3424 (5.3)	2902 (11.3)
Language spoken at home, No. (%)					
Chinese	2634 (1.8)	601 (1.2)	145 (2.8)	1570 (2.4)	318 (1.2)
English	105 117 (71.1)	38 240 (73.9)	2494 (48.0)	47 702 (73.1)	16 681 (64.8)
Spanish	25 985 (17.6)	7881 (15.2)	1930 (37.2)	10 070 (15.4)	6104 (23.7)
Missing	8476 (5.7)	2929 (5.7)	273 (5.3)	3666 (5.6)	1530 (5.9)
Other	5711 (3.9)	2104 (4.1)	351 (6.8)	2212 (3.4)	1122 (4.4)
General health rating					
Excellent	4763 (3.2)	1668 (3.2)	188 (3.6)	2172 (3.3)	735 (2.9)
Very good	14 761 (10.0)	5137 (9.9)	515 (9.9)	6757 (10.4)	2352 (9.1)
Good	45 669 (30.9)	16 224 (31.4)	1610 (31.0)	20 238 (31.0)	7597 (29.5)
Fair	58 465 (39.5)	20 205 (39.0)	2115 (40.7)	25 621 (39.3)	10 524 (40.9)
Poor	22 194 (15.0)	7835 (15.1)	685 (13.2)	9504 (14.6)	4170 (16.2)
Missing	2071 (1.4)	686 (1.3)	80 (1.5)	928 (1.4)	377 (1.5)
Comorbidities					
Any cancer	15 764 (10.7)	5555 (10.7)	468 (9.0)	6525 (10.0)	3216 (12.5)
CHF	18 946 (12.8)	6645 (12.8)	568 (10.9)	7669 (11.8)	4064 (15.8)
Diabetes^d^	54 118 (36.6)	18 403 (35.6)	1944 (37.4)	23 557 (36.1)	10 214 (39.7)
Depression	61 273 (41.4)	20 867 (40.3)	1998 (38.5)	27 804 (42.6)	10 604 (41.2)
Emphysema, asthma, or COPD	41 588 (28.1)	14 088 (27.2)	1222 (23.5)	19 191 (29.4)	7087 (27.5)
Hypertension or high blood pressure	100 262 (67.8)	35 538 (68.7)	3506 (67.5)	43 262 (66.3)	17 956 (69.7)
Stroke	17 979 (12.2)	6487 (12.5)	508 (9.8)	7201 (11.0)	3783 (14.7)
Total comorbidities, mean (SD), No.	4.2 (2.7)	4.1 (2.6)	4.1 (2.6)	4.1 (2.7)	4.6 (2.7)
PCS score, mean (SD)^e^	32.4 (12.2)	32.5 (12.3)	34.0 (12.0)	32.9 (12.2)	30.6 (11.9)
MCS score, mean (SD)^f^	45.6 (13.5)	45.7 (13.6)	45.5 (13.2)	45.4 (13.5)	46.0 (13.3)
ADL score, mean (SD)^g^	2.3 (2.8)	2.3 (2.8)	1.9 (2.5)	2.1 (2.6)	3.1 (3.1)
Quintile of ADI^h^					
1	26 155 (17.7)	9921 (19.2)	1422 (27.4)	9530 (14.6)	5282 (20.5)
2	24 689 (16.7)	7443 (14.4)	1288 (24.8)	9633 (14.8)	6325 (24.6)
3	25 136 (17.0)	7323 (14.2)	971 (18.7)	11 148 (17.1)	5694 (22.1)
4	29 083 (19.7)	9896 (19.1)	738 (14.2)	11 148 (20.8)	4881 (19.0)
5	38 879 (26.3)	15 819 (30.6)	687 (13.2)	19 495 (29.9)	2878 (11.2)
Missing	3981 (2.7)	1353 (2.6)	87 (1.7)	1846 (2.8)	695 (2.7)
Residence in health professional shortage area	6026 (4.1)	1308 (2.5)	95 (1.8)	3305 (5.1)	1318 (5.1)

Abbreviations: ADI, Area Deprivation Index; ADL, activities of daily living; CHF, congestive heart failure; COPD, chronic obstructive pulmonary disease; D-SNP, dual-eligible Special Needs Plan; ESKD, end-stage kidney disease; FIDE-SNP, fully integrated dual-eligible Special Needs Plan; GED, General Educational Development; MA, Medicare Advantage; MCS, Mental Component Summary; NR, not reported (insufficient sample size); PCS, Physical Component Summary.

^a^ Includes Cuban, Mexican, Puerto Rican, and other Hispanic.

^b^ Includes Chinese, Filipino, Indian, Japanese, Korean, Vietnamese, or other Asian.

^c^ Includes Guamanian, Hawaiian, Samoan, and other Pacific Islander.

^d^ Includes diagnosis of diabetes, high blood glucose, or sugar in urine.

^e^ For the PCS score, a higher score indicates better physical health, ie, less physical pain, fewer limitations in physical activities, and overall good physical functioning.

^f^ For the MCS score, a higher score indicates better mental health, ie, fewer psychological distress symptoms and better emotional well-being. Lower scores suggest more mental health challenges.

^g^ Range, 0 to 12; higher scores indicates decreased functional status.

^h^ Higher quintiles indicate higher ADI scores and greater socioeconomic deprivation.

In the overall sample, 51 755 beneficiaries (35.0%) were enrolled in a standard MA plan, 5193 beneficiaries (3.5%) were in a D-SNP look-alike plan, 65 220 beneficiaries (44.1%) were in a coordination-only D-SNP, and 25 755 beneficiaries (17.4%) were in a FIDE-SNP. D-SNP look-alike plans had the highest proportion of enrollees who identified as Asian (12.9%) or Hispanic (46.1%), while FIDE-SNPs had the highest proportion of White enrollees (61.9%). Overall, 59 964 respondents (40.5%) lived alone and 9618 respondents (6.5%) lived with a caregiver. A higher proportion of FIDE-SNP enrollees lived alone (45.1%) or with a caregiver (11.3%) compared with the other plan types (Table 1). A total of 67962 beneficiaries (46.0%) lived in the 40% of neighborhoods with the greatest socioeconomic disadvantage (ADI quintiles 4 and 5). However, a smaller proportion of enrollees of FIDE-SNPs (30.2%) lived in communities with the greatest socioeconomic disadvantage compared with coordination-only D-SNPs (50.7%) and standard MA plans (49.7%). Overall, FIDE-SNP beneficiaries had more comorbidities (mean [SD], FIDE-SNP: 4.6 [2.7]; coordination-only SNP: 4.1 [2.7]) vs beneficiaries with other plan types. FIDE-SNP beneficiaries had higher scores of difficulties with activities of daily living vs beneficiaries with other plan types (mean [SD], FIDE-SNP: 3.1 [3.1]; coordination-only SNP: 2.1 [2.6]).

### Bivariate Analysis: Geographic Subsample

**Figure 1.**
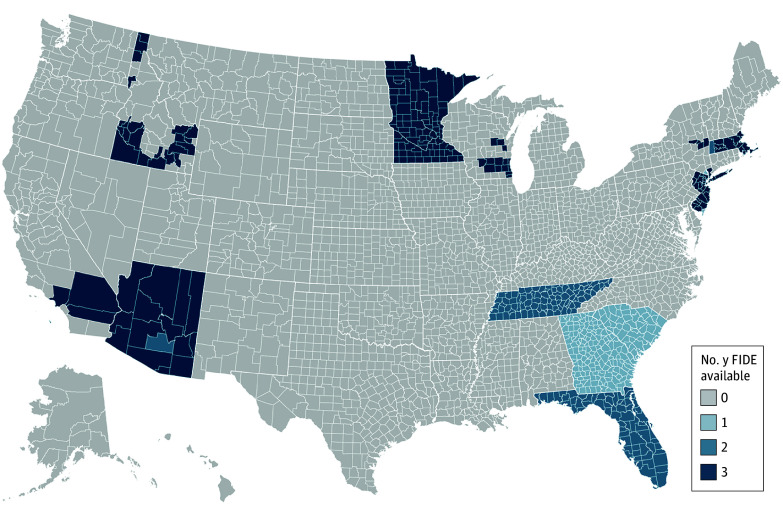
Years of Fully Integrated Dual-Eligible Special Needs Plan (FIDE-SNP) Program Availability by County, 2017-2019 The analytical subsample has been restricted to counties offering 1 or more FIDE-SNPs for each survey year. Bivariate and multivariate analyses were conducted among respondents in counties where 1 or more FIDE-SNPs were offered in a survey year.

FIDE-SNPs were available in 183 counties across 8 states in 2017, 552 counties across 12 states in 2018, and 349 counties across 10 states in 2019 (Figure 1). The geographic subsample of HOS respondents included 59 096 full-benefit dual-eligible beneficiaries in counties with at least 1 FIDE-SNP in a survey year. Characteristics of this geographic subsample are reported in Table 2.

**Table 2.** Respondent Characteristics, Dual-Eligible Beneficiaries Residing in Counties With FIDE-SNPs (Analytical Sample), 2017-2019

Characteristic	Respondents, No. (%)				
	All plan types (n = 59 096)	Standard MA (n = 11 997)	D-SNP look-alike (n = 2029)	Coordination-only D-SNP (n = 19 631)	FIDE-SNP (n = 25 439)
Age, mean (SD), y	70.1 (13.5)	69.3 (14.3)	71.4 (10.5)	65.6 (13.4)	73.9 (12.1)
Sex					
Female	37 897 (64.1)	7217 (60.2)	1198 (59.0)	12 143 (61.9)	17 339 (68.2)
Male	21 199 (35.9)	4780 (39.8)	831 (41.0)	7488 (38.1)	8100 (31.8)
Hispanic ethnicity^a^	18 281 (30.9)	4189 (34.9)	1247 (61.5)	5918 (30.2)	6927 (27.2)
Race					
Asian^b^	3985 (6.7)	807 (6.7)	348 (17.2)	1199 (6.1)	1631 (6.4)
Black or African American	12 666 (21.4)	3039 (25.3)	184 (9.1)	5236 (26.7)	4207 (16.5)
Native American	2481 (4.2)	523 (4.4)	51 (2.5)	923 (4.7)	984 (3.9)
Pacific Islander^c^	1110 (1.9)	249 (2.1)	21 (1.0)	364 (1.9)	476 (1.9)
White	32 778 (55.5)	5915 (49.30)	1007 (49.6)	10 156 (51.7)	5700 (61.7)
Missing	8720 (14.8)	2064 (17.2)	468 (23.1)	2740 (14.0)	3448 (13.6)
Education level, No. (%)					
≤Eighth grade	14 036 (23.8)	2993 (25.0)	737 (36.3)	4068 (20.7)	6238 (24.5)
Some high school, but did not graduate	9843 (16.7)	1984 (16.5)	326 (16.1)	3703 (18.9)	3830 (15.1)
High school graduate or GED	16 597 (28.1)	3202 (26.7)	366 (18.0)	5766 (29.4)	7263 (28.6)
Some college or 2-y degree	10 397 (17.6)	2120 (17.7)	237 (11.7)	3601 (18.3)	4439 (17.5)
4-y College graduate	2772 (4.7)	634 (5.3)	123 (6.1)	821 (4.2)	1194 (4.7)
>4-y College degree	1951 (3.3)	359 (3.0)	69 (3.4)	558 (2.8)	965 (3.8)
Missing	3500 (5.9)	705 (5.9)	171 (8.4)	1114 (5.7)	1510 (5.9)
Original reason for Medicare entitlement					
Age	33 852 (57.3)	6420 (53.5)	1496 (73.7)	9749 (49.7)	16 187 (63.6)
Disability	25 037 (42.4)	5537 (46.2)	529 (26.1)	9804 (49.9)	9167 (36.1)
ESKD	207 (0.4)	40 (0.3)	NR	78 (0.4)	85 (0.3)
Lives alone	24 292 (41.1)	4443 (37.0)	560 (27.6)	7783 (39.7)	11 506 (45.2)
Lives with caregiver	4346 (7.4)	797 (6.6)	79 (3.9)	643 (3.3)	2827 (11.1)
Language spoken at home					
Chinese	938 (1.6)	142 (1.2)	113 (5.6)	365 (1.9)	318 (1.3)
English	37 473 (63.4)	7578 (63.2)	485 (23.9)	12 963 (66.0)	16 447 (64.7)
Spanish	14 871 (25.2)	3114 (26.0)	1171 (57.7)	4541 (23.1)	6045 (23.8)
Missing	3434 (5.8)	731 (6.1)	113 (5.6)	1075 (5.5)	1515 (6.0)
Other	2380 (4.0)	432 (3.6)	147 (7.2)	687 (3.5)	1114 (4.4)
General health rating					
Excellent	1888 (3.2)	400 (3.3)	66 (3.3)	693 (3.5)	729 (2.9)
Very good	5817 (9.8)	1172 (9.8)	200 (9.9)	2127 (10.8)	2318 (9.1)
Good	17 973 (30.4)	3588 (29.9)	651 (32.1)	6248 (31.8)	7486 (29.4)
Fair	23 589 (39.9)	4734 (39.5)	853 (42.0)	7575 (38.6)	10 427 (41.0)
Poor	8963 (15.2)	1943 (16.2)	224 (11.0)	2691 (13.7)	4105 (16.1)
Missing	866 (1.5)	160 (1.3)	35 (1.7)	297 (1.5)	374 (1.5)
Comorbidities					
Any cancer	6560 (11.1)	1318 (11.0)	140 (6.9)	1938 (9.9)	3164 (12.4)
CHF	7865 (13.3)	1481 (12.3)	210 (10.4)	2159 (11.0)	4015 (15.8)
Diabetes^d^	22 395 (37.9)	4447 (37.1)	746 (36.8)	7105 (36.2)	10 097 (39.7)
Depression	23 877 (40.4)	4923 (41.0)	694 (34.2)	7815 (39.8)	10 446 (41.1)
Emphysema, asthma, or COPD	15 472 (26.2)	2890 (24.1)	347 (17.1)	5246 (26.7)	6989 (27.5)
Hypertension/high blood pressure	40 255 (68.1)	8159 (68.0)	1381 (68.1)	12 969 (66.1)	17 746 (69.8)
Stroke	7282 (12.3)	1459 (12.2)	159 (7.8)	1956 (10.0)	3708 (14.6)
Total comorbidities, mean (SD), No.	4.1 (2.6)	3.9 (2.6)	4.1 (2.6)	4.0 (2.6)	4.5 (2.7)
PCS score, mean (SD)^e^	32.4 (12.1)	32.0 (12.2)	35.2 (11.7)	33.6 (12.1)	30.6 (11.9)
MCS score, mean (SD)^f^	45.8 (13.4)	44.9 (13.7)	45.9 (12.8)	45.9 (13.3)	46.0 (13.3)
ADL score, mean (SD)^g^	2.5 (2.9)	2.7 (3.0)	1.8 (2.4)	1.8 (2.4)	3.1 (3.1)
ADI quintile, No. (%)^h^					
1	14 785 (25.0)	4669 (38.9)	846 (41.7)	4027 (20.5)	5243 (20.6)
2	13 403 (22.7)	3212 (26.8)	659 (32.5)	3273 (16.7)	6259 (24.6)
3	10 774 (18.2)	1497 (12.5)	270 (13.3)	3385 (17.2)	5622 (22.1)
4	10 146 (17.2)	1192 (9.9)	151 (7.4)	3995 (20.4)	4808 (18.9)
5	8468 (14.3)	1129 (9.4)	80 (3.9)	4436 (22.6)	2823 (11.1)
Missing	1520 (2.6)	298 (2.5)	23 (1.1)	515 (2.6)	684 (2.7)
Residence in health professional shortage area	1690 (2.9)	197 (1.6)	0	184 (0.9)	1309 (5.2)

Abbreviations: ADI, Area Deprivation Index; ADL, activities of daily living; CHF, congestive heart failure; COPD, chronic obstructive pulmonary disease; D-SNP, dual-eligible Special Needs Plan; ESKD, end-stage kidney disease; FIDE-SNP, fully integrated dual-eligible Special Needs Plan; GED, General Educational Development; MA, Medicare Advantage; MCS, Mental Component Summary; NR, not reported (insufficient sample size); PCS, Physical Component Summary.

^a^ Includes Cuban, Mexican, Puerto Rican, and other Hispanic.

^b^ Includes Chinese, Filipino, Indian, Japanese, Korean, Vietnamese, or other Asian.

^c^ Includes Guamanian, Hawaiian, Samoan, and other Pacific Islander.

^d^ Includes diagnosis of diabetes, high blood glucose, or sugar in urine.

^e^ For the PCS score, a higher score indicates better physical health, ie, less physical pain, fewer limitations in physical activities, and overall good physical functioning.

^f^ For the MCS score, a higher score indicates better mental health, ie, fewer psychological distress symptoms and better emotional well-being. Lower scores suggest more mental health challenges.

^g^ Range, 0 to 12; higher scores indicates decreased functional status.

^h^ Higher quintiles indicate higher ADI scores and greater socioeconomic deprivation.

Among beneficiaries aged 85 years and older, 59.0% (95% CI, 58.0%-59.9%) were enrolled in FIDE-SNPs, 22.2% (95% CI, 21.4%-23.0%) in standard MA plans, 16.1% (95% CI, 15.3%-16.8%) in coordination-only D-SNPs, and 2.8% (95% CI, 2.4%-3.1%) in D-SNP look-alike plans (Table 3). Conversely, among beneficiaries younger than age 65 years, 24.1% (95% CI, 23.6%-24.6%) were enrolled in FIDE-SNPs, 42.0% (95% CI, 41.4%-42.6%) in coordination-only D-SNPs, 30.8% (95% CI, 30.2%-31.5%) in standard MA plans, and 3.6% (95% CI, 2.7%-3.3%) in D-SNP look-alike plans. Among Black or African American beneficiaries, 38.8% (95% CI, 38.1%-39.6%) were enrolled in FIDE-SNPs, 36.9% (95% CI, 36.3%-37.8%) in coordination-only D-SNPs, and 22.2% (95% CI, 21.6%-22.8%) in standard MA plans. Conversely, 44.6% (95% CI, 44.2%-45.1%) of White beneficiaries were enrolled in FIDE-SNPs vs 30.0% (95% CI, 29.6%-30.4%) in coordination-only D-SNPs and 22.0% (95% CI, 21.5%-22.4%) in standard MA plans.

**Table 3.** Estimated Probabilities of Enrollment Across Plan Types, Beneficiaries Residing in Counties With FIDE-SNPs, Bivariate Analysis, 2017-2019^a^

Variable	Respondents, row % (95% CI)			
	Standard MA (n = 11 997)	D-SNP look-alike (n = 2029)	Coordination-only D-SNP (n = 19 631)	FIDE-SNP (n = 25 439)
Age, y				
<65	30.8 (30.2-31.5)	3.6 (2.7-3.3)	42.0 (41.4-42.6)	24.1 (23.6-24.6)
65-74	16.1 (15.7-16.5)	4.2 (4.0-4.5)	36.7 (36.2-37.2)	42.9 (42.2-43.4)
75-84	15.9 (15.4-16.5)	2.8 (2.6-3.0)	25.3 (24.7-25.9)	55.9 (55.2-56.6)
≥85	22.2 (21.4-23.0)	2.8 (2.4-3.1)	16.1 (15.3-16.8)	59.0 (58.0-59.9)
Sex				
Female	19.2 (18.9-19.6)	3.2 (3.1-3.4)	31.9 (31.5-32.2)	45.6 (45.2-46.0)
Male	22.1 (21.6-22.5)	3.7 (3.5-3.9)	35.7 (35.2-36.2)	38.5 (38.0-39.0)
Asian				
Yes	15.3 (14.5-16.1)	5.0 (4.5-5.4)	41.2 (40.1-42.3)	38.5 (37.4-39.7)
No	20.8 (20.5-21.1)	3.2 (3.1-3.3)	32.7 (32.4-33.0)	43.3 (43.0-43.6)
Black or African American				
Yes	22.2 (21.6-22.8)	2.0 (1.8-2.3)	36.9 (36.3-37.8)	38.8 (38.1-39.6)
No	20.0 (19.7-20.3)	3.7 (3.6-3.9)	32.1 (31.8-32.5)	44.2 (43.8-44.6)
Hispanic				
Yes	18.7 (18.2-19.1)	4.3 (4.1-4.5)	32.2 (31.6-32.7)	44.8 (44.2-45.4)
No	21.5 (21.1-21.8)	2.6 (2.4-2.8)	33.7 (33.3-34.0)	42.3 (41.9-42.7)
Native American				
Yes	22.3 (20.1-23.8)	2.5 (1.9-3.2)	34.8 (33.3-36.2)	40.4 (38.8-41.9)
No	20.2 (19.9-20.5)	3.5 (3.3-3.6)	33.1 (32.8-33.4)	43.2 (42.8-43.5)
Pacific Islander				
Yes	18.9 (17.1-20.6)	2.2 (1.3-3.1)	34.0 (32.0-36.1)	44.9 (42.5-47.2)
No	20.3 (20.1-20.6)	3.5 (3.3-3.6)	33.2 (32.9-33.5)	43.0 (42.7-43.3)
White				
Yes	22.0 (21.5-22.4)	3.4 (3.2-3.6)	30.0 (29.6-30.4)	44.6 (44.2-45.1)
No	19.1 (18.7-19.4)	3.4 (3.2-3.6)	36.8 (36.3-37.3)	40.7 (40.2-41.2)
Education				
Eighth grade or less	17.4 (16.9-18.0)	4.3 (4.1-4.6)	30.9 (30.3-31.5)	47.3 (46.7-48.0)
Some high school	19.7 (19.0-20.4)	3.5 (3.2-3.9)	35.5 (34.8-36.2)	41.2 (40.4-42.0)
High school graduate or GED	22.8 (22.2-23.4)	2.9 (2.7-3.2)	33.9 (33.4-34.5)	40.3 (39.7-40.9)
Some college/2 y degree	23.5 (22.8-24.3)	2.4 (2.1-2.7)	33.4 (32.7-34.1)	40.6 (39.9-41.4)
4 y college graduate	22.5 (21.2-23.8)	3.8 (3.2-4.4)	30.9 (29.5-32.3)	42.8 (41.3-44.4)
>4 y college degree	16.8 (15.8-17.7)	3.1 (2.4-3.8)	30.5 (28.7-32.2)	46.9 (45.0-48.8)
Original reason for Medicare entitlement				
Age	16.9 (16.6-17.2)	3.6 (3.4-3.8)	30.3 (29.9-30.7)	49.2 (48.7-49.6)
Disability	25.6 (25.1-26.1)	3.0 (2.8-3.2)	36.3 (35.8-36.7)	35.1 (34.6-35.6)
ESKD	25.4 (19.9-30.9)	3.8 (0.4-7.1)	28.8 (24.1-33.5)	42.1 (36.8-47.3)
Lives alone				
No	21.3 (21.0-21.7)	3.7 (3.5-3.8)	33.3 (32.9-33.7)	41.7 (41.3-42.1)
Yes	18.8 (18.4-19.3)	2.9 (2.7-3.2)	33.2 (32.7-33.6)	45.1 (44.5-45.6)
Lives with caregiver				
No	20.0 (19.7-20.3)	3.4 (3.3-3.6)	34.7 (34.4-35.0)	41.8 (41.5-42.9)
Yes	24.7 (23.4-26.0)	3.4 (2.7-4.0)	15.3 (14.3-16.2)	56.6 (55.3-58.0)
Language spoken in the home				
English	24.1 (23.7-24.5)	1.9 (1.7-2.0)	32.9 (32.5-33.2)	41.1 (40.7-41.6)
Spanish	16.5 (16.0-17.0)	4.8 (4.5-5.0)	32.3 (30.7-31.9)	47.4 (46.7-48.1)
Chinese	10.3 (8.9-11.6)	6.4 (5.4-7.4)	48.3 (46.2-50.5)	35.0 (32.9-37.1)
Other	15.7 (14.5-16.8)	5.4 (4.6-6.1)	38.4 (36.9-40.0)	40.6 (39.0-42.1)
General health rating				
Excellent	21.5 (19.9-23.1)	3.3 (2.6-4.0)	37.4 (35.7-39.0)	37.8 (36.0-39.5)
Very good	21.5 (20.5-22.4)	3.3 (2.9-3.8)	37.5 (36.6-38.5)	37.7 (36.7-38.7)
Good	20.3 (19.7-20.8)	3.4 (3.1-3.6)	36.0 (35.4-36.5)	40.4 (39.8-41.0)
Fair	19.3 (18.9-19.8)	3.6 (3.4-3.9)	32.2 (31.8-32.7)	44.8 (44.3-45.3)
Poor	21.9 (21.1-22.6)	3.0 (2.6-3.4)	27.5 (26.8-28.2)	47.6 (46.8-48.5)
High blood pressure				
Yes	19.7 (19.3-20.0)	3.4 (3.3-3.6)	31.9 (31.6-32.3)	45.0 (38.2-39.3)
No	21.7 (21.1-22.2)	3.5 (3.4-3.7)	36.1 (35.5-36.6)	38.7 (38.2-39.3)
CHF				
Yes	19.5 (18.7-20.3)	3.2 (2.8-3.6)	26.2 (25.4-27.0)	51.1 (50.2-52.0)
No	20.4 (20.1-20.7)	3.5 (3.3-3.6)	34.5 (34.2-34.8)	41.6 (41.2-42.0)
Diabetes				
Yes	19.0 (18.6-19.5)	3.3 (3.1-3.6)	31.7 (31.3-32.2)	45.9 (45.3-46.4)
No	21.2 (20.8-21.6)	3.5 (3.3-3.7)	34.1 (33.7-34.5)	41.2 (40.8-41.6)
Stroke				
Yes	21.8 (20.9-22.6)	2.8 (2.4-3.2)	24.3 (23.5-25.1)	51.1 (50.1-52.1)
No	20.1 (19.8-20.4)	3.5 (3.4-3.7)	34.7 (34.4-35.0)	41.7 (41.4-42.1)
Depression				
Yes	22.2 (21.8-22.7)	3.4 (3.2-3.6)	31.6 (31.2-32.1)	42.7 (42.2-43.2)
No	19.1 (18.7-19.4)	3.4 (3.3-3.6)	34.3 (33.9-34.7)	43.2 (42.7-43.6)
Any cancer				
Yes	21.3 (20.4-22.2)	2.5 (2.1-2.9)	29.5 (28.6-30.4)	46.7 (45.6-47.7)
No	20.4 (20.1-20.7)	3.5 (3.3-3.6)	33.6 (33.2-33.9)	42.6 (42.2-42.9)
Emphysema, asthma, or COPD				
Yes	20.4 (19.8-21.0)	2.9 (2.6-3.2)	32.1 (31.5-32.7)	44.6 (44.0-45.3)
No	20.3 (20.0-20.7)	3.6 (3.4-3.7)	33.7 (33.3-34.0)	42.4 (42.0-42.8)
Quintile of ADL Summary score^b^				
1	18.9 (18.6-19.3)	3.6 (3.4-3.7)	40.3 (39.9-40.7)	37.2 (36.8-37.6)
2	20.3 (19.7-20.9)	3.2 (2.9-3.5)	28.0 (27.4-28.6)	48.5 (47.8-49.2)
3	22.4 (21.4-23.4)	3.3 (2.7-3.8)	21.4 (20.5-22.4)	52.9 (51.8-54.1)
4	25.2 (23.8-26.7)	3.3 (2.5-4.1)	14.2 (13.1-15.3)	57.2 (55.6-58.8)
5	28.3 (26.1-30.4)	1.8 (0.9-2.7)	11.7 (10.2-13.1)	58.2 (56.0-50.5)
Quintile of ADI^c^				
1	20.6 (20.1-21.1)	4.3 (3.9-4.5)	32.0 (31.3-32.8)	43.2 (42.4-43.9)
2	20.3 (19.8-20.9)	3.8 (3.5-4.1)	30.5 (29.8-31.2)	45.4 (44.7-46.1)
3	20.7 (19.9-21.6)	2.9 (2.6-3.2)	32.2 (31.4-33.0)	44.2 (43.3-45.0)
4	20.6 (19.6-21.6)	2.7 (2.3-3.1)	34.4 (33.6-35.2)	42.3 (41.3-43.2)
5	18.5 (17.4-19.7)	1.8 (1.4-2.1)	38.4 (37.5-39.3)	41.3 (40.2-42.4)
Quintile of PCS score^d^				
1	21.5 (21.0-22.0)	2.7 (2.5-3.0)	26.2 (25.7-26.7)	49.5 (48.9-50.1)
2	19.5 (19.0-20.1)	3.6 (3.3-3.9)	32.6 (32.0-33.1)	44.3 (43.7-45.0)
3	19.3 (18.7-19.9)	3.7 (3.4-4.0)	37.8 (37.2-38.5)	39.3 (38.6-40.0)
4	19.9 (19.1-20.6)	3.7 (3.3-4.0)	40.2 (39.4-41.0)	36.2 (35.4-37.0)
5	20.3 (19.3-21.3)	4.1 (3.6-4.5)	43.9 (42.9-44.9)	31.7 (30.7-32.7)
Quintile of MCS score^e^				
1	21.7 (21.2-22.2)	3.6 (3.3-3.8)	31.5 (31.0-32.0)	43.2 (42.7-43.8)
2	19.4 (18.9-20.0)	3.7 (3.4-4.0)	33.4 (32.8-34.0)	43.5 (42.8-44.1)
3	19.9 (19.2-20.6)	3.2 (2.8-3.5)	34.4 (33.7-35.1)	42.5 (41.7-43.3)
4	19.1 (18.2-20.0)	3.4 (2.9-3.8)	35.9 (34.9-36.8)	41.7 (40.7-42.7)
5	19.3 (18.6-20.1)	3.0 (2.7-3.4)	34.3 (33.5-35.1)	43.4 (42.4-44.3)
Residence in health professional shortage area				
Yes	24.1 (19.6-28.5)	NR	25.5 (23.1-28.0)	50.4 (46.5-54.3)
No	20.3 (20.0-20.6)	3.4 (3.3-3.6)	33.4 (33.1-33.7)	42.9 (42.6-43.2)

Abbreviations: ADI, Area Deprivation Index; ADL, activities of daily living; CHF, congestive heart failure; COPD, chronic obstructive pulmonary disease; D-SNP, dual-eligible Special Needs Plan; ESKD, end-stage kidney disease; FIDE-SNP, fully integrated dual-eligible Special Needs Plan; GED, General Educational Development; MA, Medicare Advantage; MCS, Mental Component Summary; NR, not reported (insufficient sample size).

^a^ Bivariate analysis examines the association between respondent characteristics and enrollment in each MA plan type, considering each respondent characteristic one at a time. Estimates adjusted for state-fixed effects. Reported are predicted margins, which represent the probability that an individual with a given characteristic (eg, age ≥85 years) is enrolled in one plan type, compared with all other plan types. Estimates are differences in predicted probabilities on 0 to 100 percentage point scale. Analysis in a geographic subsample of respondents to the Health Outcomes Survey with dual eligibility for Medicare and full Medicaid and residence in counties with at least 1 FIDE-SNP in the survey years (59 096 dual-eligible Health Outcomes Survey respondents).

^b^ Higher quintiles are associated with decreased functional status.

^c^ Higher quintiles are associated with higher ADI scores and greater socioeconomic deprivation.

^d^ Higher quintiles are associated with greater physical functioning.

^e^ Higher quintiles are associated with greater mental functioning.

Among beneficiaries rating their general health as poor, 47.6% (95% CI, 46.8%-48.5%) were enrolled in FIDE-SNPs, 27.5% (95% CI, 26.8%-28.2%) in coordination-only D-SNPs, and 21.9% (95% CI, 21.1%-22.6%) in standard MA plans. Higher proportions of beneficiaries with chronic conditions, including congestive heart failure, hypertension, and stroke, were enrolled in FIDE-SNPs or coordination-only D-SNPs vs other plan types (eg, among those with a history of stroke, 51.1% [95% CI, 50.1%-52.1%] were enrolled in FIDE-SNPs, 24.3% [95% CI, 23.5%-25.1%] in coordination-only D-SNPs, and 21.8% [95% CI, 20.9%-22.6%] in standard MA). Fully 56.6% (95% CI, 55.3%-58.0%) of beneficiaries living with a paid caregiver and 45.1% (95% CI, 44.5%-45.6%) of those living alone were enrolled in FIDE-SNPs, while 15.3% (95% CI, 14.3%-16.2%) of beneficiaries living with a caregiver and 33.2% (95% CI, 32.7%-33.6%) of those living alone were enrolled in coordination-only D-SNPs. Fully 58.2% (95% CI, 56.0%-50.5%) of beneficiaries with the greatest frailty (highest ADL quintile) were enrolled in FIDE-SNPs, compared with 11.7% (95% CI, 10.2%-13.1%) in coordination-only D-SNPs.

**Figure 2.**
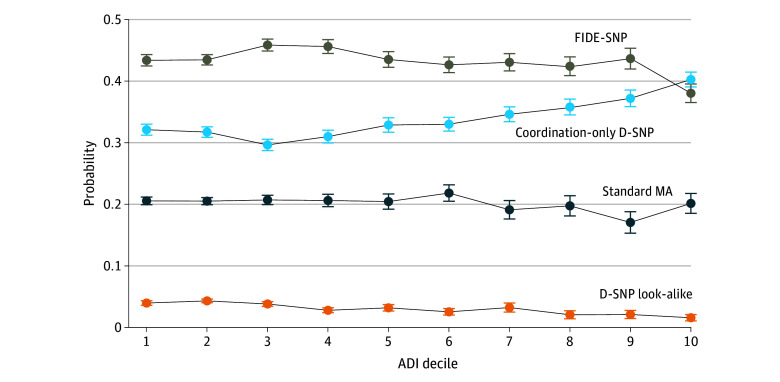
Estimated Probability of Enrollment by Plan Type Across Area Disadvantage Index (ADI) Deciles Among Beneficiaries Residing in Counties With Fully Integrated Dual-Eligible Special Needs Plans (FIDE-SNPs), 2017-2019 Bivariate analysis was used to examine the association between respondent characteristics and enrollment in each Medicare Advantage (MA) plan type, analyzing each characteristic individually. The estimated margins for ADI decile are shown, comparing the probability that an individual residing in an area with a specific level of deprivation is enrolled in 1 plan type vs all other plan types. Whiskers indicate 95% CIs. Analysis in a geographic subsample of respondents to the Health Outcomes Survey with dual eligibility for Medicare and Medicaid and residence in counties with at least 1 FIDE-SNP in the survey years (59 096 full-benefit dual-eligible Health Outcomes Survey respondents). D-SNP indicates dual-eligible Special Needs Plan.

Among people living in the most socially disadvantaged neighborhoods (ADI quintile 5), 38.4% (95% CI, 37.5%-39.3%) were enrolled in coordination only D-SNPs vs 41.3% (95% CI, 40.2%-42.4%) in FIDE-SNPs. Among respondents living in the least socially disadvantaged neighborhoods (ADI quintile 1), 32.0% (95% CI, 31.3%-32.8%) were enrolled in coordination-only D-SNPs vs 43.2% (95% CI, 42.4%-43.9%) in FIDE-SNPs (Table 3). This association was most pronounced in the 10% most disadvantaged neighborhoods, where FIDE-SNPs enrolled proportionately fewer individuals and coordination-only D-SNPs enrolled proportionately more (Figure 2).

### Multivariate Analyses

Estimates from the multivariate model show that, although the differences in enrollee characteristics across plan types were somewhat smaller, most patterns observed in the bivariate model remained consistent (eTable in Supplement 1). Older beneficiaries and those living alone or with a paid caregiver were more likely to enroll in FIDE-SNPs vs other plan types. However, differences in the probability of enrolling in FIDE-SNPs vs coordination-only D-SNPs or standard MA plans were less pronounced by racial and ethnic groups. Similar to the bivariate analysis, the multivariate analysis found that among beneficiaries with the greatest frailty (highest ADL score quintile), 53.9% (95% CI, 51.6%-56.1%) were enrolled in FIDE-SNPs, while among those with the lowest frailty (lowest ADL score quintile), 39.5% (95% CI, 39.1%-39.9%) were enrolled in FIDE-SNPs.

## Discussion

This national, cross-sectional study of full-benefit dual-eligible beneficiaries enrolled in MA found high levels of patient-reported chronic disease burden and functional limitations overall, with several notable differences across MA plans attaining different levels of integration with Medicaid. First, compared with less integrated plans, FIDE-SNPs served beneficiaries who were older and more functionally impaired. This may be partially accounted for by policies in certain states, such as Massachusetts, that limited FIDE-SNP enrollment to dual-eligible beneficiaries ages 65 years and older (younger dual-eligible individuals could enroll in the state’s Medicare-Medicaid Plans).^24^ Second, FIDE-SNPs enrolled a higher proportion of beneficiaries eligible for full Medicaid, White beneficiaries, and those living alone or with a paid caregiver. Third, the proportion of dual-eligible beneficiaries residing in the most socially disadvantaged neighborhoods who enrolled in FIDE-SNPs was somewhat lower than the proportion living in the least disadvantaged neighborhoods. Altogether, findings highlight salient differences in enrollee characteristics across MA plan types and add to evidence of this population’s medical and social vulnerability.

Dual-eligible beneficiaries have a high prevalence of physical and behavioral health care needs and social risk factors linked to their age or presence of a disability (basis of Medicare eligibility) and low-income status (basis of Medicaid eligibility).^19,25,26,27,28^ There are longstanding concerns that a lack of coordination across Medicare and Medicaid results in a poorly configured system for addressing these needs.^29^ Such concerns have prompted national efforts to expand integrated care programs in which one entity—typically a managed care plan—manages both Medicare and Medicaid benefits and spending. The increase in D-SNPs that specialize in care for dual-eligible beneficiaries and the expansion of Medicaid managed care programs with long-term care coverage have provided opportunity to expand integrated care plans. Although FIDE-SNPs (which bear risk for both Medicare and Medicaid spending) are only offered in a few states and cover a small proportion of dual-eligible beneficiaries, policymakers continue to focus on these plans as a framework for expanding integrated care.^29,30^

To our knowledge, this is the first study to examine differences in patient-reported characteristics across MA plan types attaining different levels of integration. A previous study used administrative data to describe the characteristics of D-SNP enrollees with varying levels of Medicaid integration but did not include other MA plans or incorporate respondent-reported data on health, socioeconomic status, or living circumstances.^31^ Our finding of substantial health and social vulnerability among dual-eligible enrollees across a range of MA plan types has several policy implications. The first relates to the evolving landscape of integrated plans, increases in MA enrollment, and expansion of plan variety. Policymakers are concerned about whether dual-eligible beneficiaries are enrolling in plans equipped to coordinate care for their complex medical, long-term care, and social needs. While FIDE-SNPs served populations with somewhat greater functional impairment, we found a high prevalence of medical and social risk factors across all MA plan types, including D-SNP look-alike plans, raising concerns because look-alike plans are not subject to any of the D-SNP requirements to coordinate care.

Second, after controlling for geographic differences in the availability of FIDE-SNPs, these plans tended to serve fewer beneficiaries living in the most socioeconomically disadvantaged neighborhoods and a higher proportion of White beneficiaries. Prior studies found higher-quality MA plans are less likely to be offered in areas with higher rates of Black and Hispanic beneficiaries. Those studies concluded that disparities were explained, in part, by the lower availability of highly rated MA plans in areas with a high share of Black or Hispanic Medicare beneficiaries and greater socioeconomic vulnerability.^32,33^ In this study, differences in FIDE-SNP enrollment by race and ethnicity and community-level socioeconomic status persisted after limiting to counties where the plans were offered, suggesting factors other than plan availability may contribute to enrollment differences. Therefore, understanding the factors associated with socially at-risk dual-eligible enrollment in FIDE-SNPs warrants further attention.

Third, our results highlight the need to account for differences in beneficiaries’ health, demographic, and social characteristics in risk adjustment to compare plan performance.^25,34^ This can be challenging because some characteristics, including functional status and social supports, are not captured directly in administrative datasets commonly used in research. Linking survey data with administrative data provides a comprehensive picture of these characteristics. Additionally, findings underscore the need to explore how care and outcomes vary among dual-eligible beneficiaries with different health and social needs.

### Limitations

This study had several limitations. First, our analysis was limited to full-benefit dual-eligible beneficiaries enrolled in MA plans, excluding traditional Medicare beneficiaries. Although MA enrollment has increased, approximately half of dual-eligible beneficiaries remain enrolled in traditional Medicare. Second, this study sought to examine the association of health status and beneficiary characteristics with MA plan type but could not establish causal relationships. Third, survey analysis may introduce nonresponse bias if respondents differ from nonrespondents, but the HOS is ideal for this analysis because it has the largest response rate among surveys for this population. Fourth, because FIDE-SNPs were only available in certain counties, we analyzed county-years with at least 1 FIDE-SNP, though these counties did not always overlap with those offering D-SNP look-alikes.

## Conclusions

This cross-sectional study highlights the substantial differences in characteristics among full-benefit dual-eligible beneficiaries enrolled in various MA plan types. We found that while these beneficiaries had substantial health and social risk factors overall, those enrolled in FIDE-SNPs were generally older, had higher levels of frailty and comorbidities, and were more likely to live alone or with a paid caregiver compared with those in other MA plans types. However, FIDE-SNP enrollees tended to live in less socioeconomically deprived areas. These findings underscore the importance of tailoring policy efforts to address the unique needs of different dual-eligible populations within MA and highlight the need for further research to understand the factors influencing MA plan choices among dual-eligible beneficiaries.

## Notes

Supplement 1. **eTable.** Analytical Sample Multivariate Margins

Supplement 2.
Data Sharing Statement
