# Supplementary material for: Patient-Reported Characteristics Across Dual-Eligible Medicare Advantage Plan Types
Source: JAMA Netw Open. 2025 Apr 18;8(4):e255791. doi: 10.1001/jamanetworkopen.2025.5791 (PMC12008755; doi:10.1001/jamanetworkopen.2025.5791)
Supplement: Supplement 1. — eTable. Analytical Sample Multivariate Margins [file jamanetwopen-e255791-s001.pdf]

Supplemental Online Content

Offiaeli K, Meyers DJ, Macneal E, Johnston KJ, Brown-Podgorski B, Roberts ET. Patient-reported characteristics across dual-eligible Medicare Advantage plan types. *JAMA Netw Open*. 2025;8(4):e255791. doi:10.1001/jamanetworkopen.2025.5791

eTable. Multivariate predicted probabilities of enrollment across plan types, 2017-2019

This supplemental material has been provided by the authors to give readers additional information about their work.

© 2025 Offiaeli K et al. JAMA Network Open.

eTable: Multivariate predicted probabilities of enrollment across plan types, 2017-2019

Variable	Standard MA n=11997	D-SNP "Look-alike" n=2029	Coordination-only D-SNP n=19631	FIDE-SNP n=25439
Age Group				
<65	27.4 (26.5 - 28.2)	4.2 (3.6 - 4.8)	43.4 (42.6 - 44.2)	25.1 (24.4 - 25.7)
65-74	17.0 (16.5 - 17.4)	4.1 (3.8 - 4.3)	34.3 (33.8 - 34.8)	44.7 (44.1 - 45.2)
75-84	16.9 (16.4 - 17.4)	2.6 (2.4 - 2.8)	25.6 (24.9 - 26.2)	54.9 (54.2 - 55.6)
85+	22.8 (21.9 - 23.7)	2.7 (2.3 - 3.0)	19.7 (18.8 - 20.6)	54.8 (53.8 - 55.9)
Sex				
Female	19.6 (19.3 - 20.0)	3.3 (3.2 - 3.5)	33.2 (32.9 - 33.5)	43.9 (43.5 - 44.2)
Male	21.5 (21.1 - 22.0)	3.6 (3.4 - 3.8)	33.3 (32.9 - 33.8)	41.6 (41.0 - 42.1)
Asian				
Yes	18.8 (17.1 - 20.5)	4.8 (3.6 - 6.0)	36.4 (34.6 - 38.3)	40.0 (38.0 - 42.0)
No	20.5 (20.1 - 20.8)	3.2 (3.1 - 3.4)	33.0 (32.7 - 33.3)	43.3 (43.0 - 43.7)
Black/African American				
Yes	20.7 (19.4 - 22.0)	3.2 (2.3 - 4.0)	35.9 (34.6 - 37.2)	40.2 (38.7 - 41.8)
No	20.3 (19.8 - 20.8)	3.5 (3.3 - 3.6)	32.4 (31.9 - 32.9)	43.8 (43.3 - 44.3)
Hispanic				
Yes	20.7 (20.1 - 21.3)	3.8 (3.6 - 4.1)	33.7 (33.0 - 34.3)	41.8 (41.1 - 42.5)
No	20.1 (19.7 - 20.6)	2.9 (2.7 - 3.2)	33.1 (32.7 - 33.5)	43.8 (43.3 - 44.3)
Native American				
Yes	19.8 (18.4 - 21.2)	3.6 (2.6 - 4.5)	33.9 (32.5 - 35.2)	42.8 (41.3 - 44.3)
No	20.3 (20.0 - 20.6)	3.4 (3.3 - 3.6)	33.2 (32.9 - 33.5)	43.1 (42.8 - 43.4)
Pacific Islander				
Yes	20.2 (18.1 - 22.3)	1.9 (1.0 - 2.8)	34.5 (32.3 - 36.6)	43.4 (40.9 - 46.0)
No	20.3 (20.0 - 20.6)	3.5 (3.3 - 3.6)	33.2 (32.9 - 33.5)	43.0 (42.7 - 43.3)
White				
Yes	21.6 (20.7 - 22.6)	3.2 (2.7 - 3.6)	32.3 (31.5 - 33.1)	42.9 (42.0 - 43.8)
No	19.2 (18.4 - 20.0)	3.7 (3.2 - 4.3)	34.0 (33.1 - 34.9)	43.1 (42.0 - 44.2)
Education				
8th Grade or Less	20.1 (29.6 - 20.7)	3.4 (3.2 - 3.7)	34.1 (33.5 -34.6)	42.3 (41.7 - 43.0)
Some High School	19.6 (18.9 - 20.2)	3.8 (3.4 - 4.1)	34.5 (33.9 - 35.2)	42.1 (41.4 - 42.9)
High School Graduate/GED	20.7 (20.2 - 21.3)	3.5 (3.2 - 3.8)	33.0 (32.4 - 33.5)	42.8 (42.2 - 43.4)

© 2025 Offiaeli K et al. JAMA Network Open.

Some College/2-yr degree	20.9 (20.2 - 21.6)	3.2 (2.8 - 3.6)	32.2 (31.5 - 32.9)	43.7 (42.9 - 44.4)
4-yr college graduate	22.4 (21.1 - 23.7)	3.5 (2.9 - 4.1)	30.2 (29.0 - 31.4)	44.0 (42.5 - 45.4)
> 4-yr college degree	19.8 (18.3 - 21.3)	3.3 (2.6 - 4.1)	30.9 (29.5 - 32.4)	45.9 (44.2 - 47.6)
Original Entitlement Reason				
Age	19.6 (19.2 - 20.0)	3.4 (3.3 - 3.6)	34.5 (34.1 - 34.9)	42.5 (42.0 - 42.9)
Disability	21.2 (20.6 - 21.7)	3.4 (3.1 - 3.8)	31.7 (31.1 - 32.2)	43.7 (43.1 - 44.3)
ESRD	19.1 (14.5 - 23.7)	3.3 (0.4 - 6.1)	23.0 (19.1 - 26.8)	54.6 (49.3 - 60.0)
Lives Alone				
No	21.2 (20.9 - 21.6)	3.4 (3.2 - 3.6)	33.7 (33.4 - 34.1)	41.6 (41.2 - 42.0)
Yes	18.9 (18.5 - 19.4)	3.5 (3.3 - 3.8)	32.6 (32.1 - 33.0)	45.0 (44.5 - 45.5)
Lives w/Caregiver				
No	20.2 (19.9 - 20.5)	3.4 (3.5 - 3.6)	33.9 (33.6 - 34.2)	42.5 (42.2 - 42.8)
Yes	22.1 (20.9 - 23.3)	4.2 (3.4 - 5.0)	23.1 (22.0 - 24.3)	50.6 (49.3 - 51.9)
Language Spoken in the Home				
English	23.2 (22.7 - 23.7)	2.0 (1.8 - 2.2)	32.6 (32.2 - 33.0)	42.3 (41.7 - 42.8)
Spanish	17.0 (16.4 - 17.6)	4.9 (2.5 - 5.3)	33.0 (32.2 - 33.8)	45.1 (44.3 - 46.0)
Chinese	13.8 (12.0 - 15.7)	4.4 (3.4 - 5.4)	45.9 (45.6 - 48.2)	35.8 (33.5 - 38.2)
Other	18.0 (16.7 - 19.3)	4.2 (3.4 - 5.0)	38.2 (36.7 - 39.7)	39.6 (38.0 - 41.1)
General Health Rating				
Excellent	21.0 (19.3 - 22.6)	3.2 (2.5 - 4.0)	30.2 (28.6 - 31.7)	45.6 (43.7 - 47.5)
Very Good	21.2 (20.2 - 22.2)	3.4 (2.9 - 3.8)	32.3 (31.3 - 33.2)	43.1 (42.0 - 44.2)
Good	20.8 (20.2 - 21.3)	3.3 (3.1 - 3.6)	32.9 (32.4 - 33.5)	43.0 (42.4 - 43.6)
Fair	19.8 (19.4 - 20.3)	3.6 (3.3 - 3.8)	33.4 (33.0 - 33.8)	43.2 (42.7 - 43.7)
Poor	20.1 (19.3 - 20.8)	3.3 (2.9 - 5.0)	34.3 (33.5 - 35.0)	42.3 (41.5 - 43.2)
High Blood Pressure				
Yes	20.3 (19.9 - 20.6)	3.5 (3.3 - 3.7)	33.2 (32.9 - 33.5)	43.0 (42.7 - 43.4)
No	20.4 (19.9 - 20.9)	3.3 (3.0 - 3.5)	33.3 (32.8 - 33.8)	43.1 (42.5 - 43.7)
Congestive Heart Failure				
Yes	20.1 (19.3 - 20.9)	3.5 (3.1 - 3.9)	32.2 (31.5 - 33.0)	44.2 (43.3 - 45.0)
No	20.3 (20.0 - 20.6)	3.3 (3.0 - 3.6)	33.4 (33.1 - 33.7)	42.9 (42.5 - 43.2)
Diabetes				
Yes	19.7 (19.3 - 20.1)	3.2 (3.0 - 3.4)	32.9 (32.5 - 33.3)	44.2 (43.7 - 44.7)
No	20.7 (20.4 - 21.1)	3.3 (3.0 - 3.5)	33.4 (33.1 - 33.8)	42.2 (41.9 - 42.6)
Stroke				
Yes	21.0 (20.2 - 21.9)	3.4 (2.9 - 3.9)	28.7 (28.9 - 30.5)	45.9 (45.0 - 46.8)
No	20.2 (19.9 - 20.5)	3.4 (3.3 - 3.6)	33.8 (33.5 - 34.1)	42.6 (42.2 - 42.9)
Depression				
Yes	20.8 (20.3 - 21.3)	3.4 (3.1 - 3.6)	32.7 (32.2 - 33.2)	43.1 (42.6 - 43.7)
No	20.0 (19.6 - 20.4)	3.4 (3.3 - 3.6)	33.6 (33.2 - 33.9)	43.0 (42.6 - 43.4)
Any Cancer				
Yes	21.5 (20.6 - 22.4)	2.8 (3.1 - 3.6)	33.1 (32.3 - 33.9)	42.6 (41.7 - 43.5)
No	20.3 (20.0 - 20.6)	3.4 (3.3 - 3.6)	33.1 (32.8 - 33.4)	43.1 (42.8 - 43.5)

© 2025 Offiaeli K et al. JAMA Network Open.

Emphysema, Asthma, or COPD				
Yes	19.5 (18.9 - 20.0)	3.5 (3.1 - 3.8)	33.4 (32.9 - 33.9)	43.6 (43.0 - 44.3)
No	20.6 (20.3 - 20.9)	3.4 (3.3 - 3.6)	33.1 (32.8 - 33.4)	42.8 (42.5 - 43.2)
ADL Score Quintile				
1	19.3 (18.9 - 19.7)	3.4 (3.2 - 3.6)	37.8 (37.4 - 38.2)	39.5 (39.1 - 39.9)
2	20.4 (19.8 - 21.0)	3.6 (3.2 - 3.9)	29.6 (29.0 - 30.2)	46.4 (45.7 - 47.1)
3	22.7 (21.7 - 23.)	3.6 (3.0 - 4.2)	24.9 (24.0 - 25.9)	48.8 (47.7 - 49.9)
4	24.5 (23.0 - 25.9)	4.1 (3.1 - 5.0)	19.5 (18.2 - 20.8)	51.9 (50.4 - 53.5)
5	27.0 (25.0 - 29.1)	2.2 (1.1 - 3.2)	16.9 (15.2 - 18.7)	53.9 (51.6 - 56.1)
PCS Quintile				
1	20.3 (19.8 - 20.9)	3.0 (2.7 - 3.3)	31.3 (30.7 - 31.8)	45.4 (44.8 - 46.0)
2	19.9 (19.3 - 20.4)	3.6 (3.3 - 3.8)	32.6 (32.1 - 33.1)	44.0 (43.4 - 44.6)
3	20.5 (19.9 - 21.1)	3.5 (3.2 - 3.8)	34.5 (33.9 - 35.1)	41.5 (40.9 - 42.2)
4	21.0 (20.2 - 21.9)	3.5 (3.2 - 3.9)	35.1 (34.3 - 35.9)	40.4 (39.5 - 41.3)
5	20.6 (19.5 - 21.7)	3.9 (3.4 - 4.4)	37.3 (36.2 - 38.4)	38.2 (37.0 - 39.4)
MCS Quintile				
1	20.2 (19.7 - 20.8)	3.6 (3.3 - 3.9)	33.9 (33.3 - 34.4)	42.3 (41.7 - 42.9)
2	20.1 (19.6 - 20.6)	3.6 (3.3 - 3.9)	33.0 (32.5 - 33.5)	43.3 (42.7 - 43.9)
3	20.7 (20.0 - 21.4)	3.2 (2.8 - 3.5)	32.8 (32.1 - 33.4)	43.4 (42.6 - 44.1)
4	20.2 (19.2 - 21.1)	3.4 (3.0 - 3.8)	32.9 (32.0 - 33.8)	43.6 (42.5 - 44.6)
5	20.4 (19.5 - 21.2)	3.0 (2.7 - 3.4)	32.8 (32.0 - 33.6)	43.8 (42.9 - 44.7)
Residence in Health Professional Shortage Area				
Yes	23.7 (19.7 - 27.7)	— (a)	33.3 (26.4 - 30.9)	47.7 (44.3 - 51.1)
No	20.3 (20.0 - 20.6)	3.4 (3.3 - 3.6)	33.3 (33.1 - 33.6)	43.0 (42.7 - 43.3)

Multivariate analysis examines the association between respondent characteristics and 350 enrollment in each MA plan type, considering all respondent characteristics simultaneously. Estimates adjusted for state fixed effects. Reported are predicted margins, which compare the probability that an individual with a given characteristic (e.g., age >85 years or enrollment in full Medicaid) is enrolled in one plan type, compared to all other plan types, holding state and all other respondent characteristics constant. Estimates are differences in predicted probabilities on 0-100 point scale. Analysis in a geographic subsample of respondents to the Health Outcomes Survey with dual eligibility for Medicare and full Medicaid and residence in counties with at least one FIDE-SNP in the survey year (n=59096 dual-eligible HOS respondents).
(a) Not reported due to insufficient sample size.

© 2025 Offiaeli K et al. JAMA Network Open.
