# Supplementary material for: Patient-Reported Characteristics Across Dual-Eligible Medicare Advantage Plan Types
Source: JAMA Netw Open. 2025 Apr 18;8(4):e255791. doi: 10.1001/jamanetworkopen.2025.5791 (PMC12008755; doi:10.1001/jamanetworkopen.2025.5791)
Supplement: Supplement 2. — Data Sharing Statement [file jamanetwopen-e255791-s002.pdf]

Data Sharing Statement

Offiaeli. Patient-Reported Characteristics Across Dual-Eligible Medicare Advantage Plan Types. *JAMA Netw Open*. Published April 18, 2025. doi:10.1001/jamanetworkopen.2025.5791

Data
Data available: No
